# Novel loci linked to serum lipid traits are identified in a genome-wide association study of a highly admixed Brazilian population - the 2015 ISA Nutrition

**DOI:** 10.1186/s12944-024-02085-1

**Published:** 2024-07-26

**Authors:** Jean Michel R. S. Leite, Jaqueline L. Pereira, Camila Alves de Souza, Júlia M. Pavan Soler, Regina Célia Mingroni-Netto, Regina M. Fisberg, Marcelo M. Rogero, Flavia M. Sarti

**Affiliations:** 1 School of Public Health, University of São Paulo, São Paulo, Brazil; 2 Institute of Mathematics and Statistics, University of São Paulo, São Paulo, Brazil; 3 School of Arts, Sciences and Humanities, University of São Paulo, São Paulo, Brazil; 4 Institute of Biosciences, University of São Paulo, São Paulo, Brazil

**Keywords:** Dyslipidemia, Genomics, Lipoproteins/Metabolism, Lipids, Lipidomics

## Abstract

**Background:**

Cardiovascular diseases (CVDs) comprise major causes of death worldwide, leading to extensive burden on populations and societies. Alterations in normal lipid profiles, i.e., dyslipidemia, comprise important risk factors for CVDs. However, there is lack of comprehensive evidence on the genetic contribution to dyslipidemia in highly admixed populations. The identification of single nucleotide polymorphisms (SNPs) linked to blood lipid traits in the Brazilian population was based on genome-wide associations using data from the São Paulo Health Survey with Focus on Nutrition (ISA-Nutrition).

**Methods:**

A total of 667 unrelated individuals had genetic information on 330,656 SNPs available, and were genotyped with Axiom™ 2.0 Precision Medicine Research Array. Genetic associations were tested at the 10^− 5^ significance level for the following phenotypes: low-density lipoprotein cholesterol (LDL-c), very low-density lipoprotein cholesterol (VLDL-c), high-density lipoprotein cholesterol (HDL-c), HDL-c/LDL-c ratio, triglycerides (TGL), total cholesterol, and non-HDL-c.

**Results:**

There were 19 significantly different SNPs associated with lipid traits, the majority of which corresponding to intron variants, especially in the genes *FAM81A*, *ZFHX3*, *PTPRD*, and *POMC*. Three variants (rs1562012, rs16972039, and rs73401081) and two variants (rs8025871 and rs2161683) were associated with two and three phenotypes, respectively. Among the subtypes, non-HDL-c had the highest proportion of associated variants.

**Conclusions:**

The results of the present genome-wide association study offer new insights into the genetic structure underlying lipid traits in underrepresented populations with high ancestry admixture. The associations were robust across multiple lipid phenotypes, and some of the phenotypes were associated with two or three variants. In addition, some variants were present in genes that encode ncRNAs, raising important questions regarding their role in lipid metabolism.

**Supplementary Information:**

The online version contains supplementary material available at 10.1186/s12944-024-02085-1.

## Background

Cardiovascular diseases (CVDs) comprise major causes of death worldwide, resulting in extensive burden of early mortality, reduction in quality of life, and other socioeconomic and health impacts on populations and societies [1, 2]. Alterations in normal lipid profiles, i.e., dyslipidemia, are risk factors significantly associated with CVD considering the mechanisms linked to the pathophysiology of atherosclerosis [3, 4]. However, comprehensive evidence on the relationships between dyslipidemia and other CVD risk factors is lacking, considering that only part of the variance in lipid traits is explained by traditional risk factors (e.g., lifestyle, demographic and socioeconomic characteristics, biochemical mechanisms, among others). Heritability, candidate genes, and genome-wide association studies (GWASs) have been performed to fill the gap in the literature, revealing the considerable genetic influence on lipid traits [5–7].

However, a major part of genetic investigations has been conducted in European descent populations, which hinders the extrapolation of findings to groups of admixed ancestry [8]. In fact, a recent analysis showed that the power of GWASs might be increased using data from admixed populations [9]. A study performed in the Brazilian population comprising a mix of multiple ancestries estimated moderate heritabilities for LDL-c, HDL-c, total cholesterol, and triglycerides (TGL) in a family-based investigation [10]. Other studies in Brazil have identified links between single nucleotide polymorphisms (SNPs) and fatty acid profiles and serum lipid traits under the candidate-gene framework [11–13].

The Sao Paulo Health Survey with Focus on Nutrition (ISA-Nutrition) represents one of the pioneering initiatives in Brazil inquiring about the relationship between dyslipidemia and CVD risk factors, including SNPs [14]. While the studies performed using ISA-Nutrition data provided initial insights, the genetic contribution to dyslipidemia and its underlying mechanisms remains to be fully understood [7]. For instance, these previous studies rely on *a priori* hypothesis over a limited set of markers, which impacts on the discovery of potential novel variants throughout the genome [15]. Therefore, the present study aimed to perform a genome-wide association study (GWAS) to detect SNPs linked to blood lipid traits in individuals participating in the ISA-Nutrition study, assuming a linear additive genetic model. The hypothesis of the study refers to the existence of diverse genetic contributions to lipid traits within highly-admixed populations, representing novel evidence regarding the role of genetic information from individuals in underexplored ethnic groups.

## Materials and methods

### Study design and population

The present study is part of the cross-sectional population-based Sao Paulo Health Survey with Focus on Nutrition study (ISA Nutrition), conducted in 2015, which aims to investigate the associations of lifestyle, sociodemographic, economic, biochemical, and genetic information with cardiometabolic diseases in the city of São Paulo. The present study was conducted in accordance with the principles of the Declaration of Helsinki, being approved by the Research Ethics Committee of the School of Public Health from the University of São Paulo (43838621.7.0000.5421 and 30848914.7.0000.5421). The details of the study are described elsewhere [14].

Data initially comprised information collected from 901 residents in São Paulo municipality during 2015. Participants were distributed in three groups according to age: adolescents (corresponding to individuals ≥ 12 to 19 years old), adults (individuals ≥ 20 to 59 years old), and older adults (individuals ≥ 60 years old). Questionnaires were administered by trained personnel, including information on socioeconomic, demographic, anthropometric, lifestyle, and health status of individuals, among other characteristics. Blood pressure, anthropometric data and blood samples were collected from the participants in the households by trained nurses for the identification of biochemical and genetic markers. Further details on the sampling procedure and summary statistics of this dataset were previously described in other publications [7, 14, 16].

### Phenotypic data

Previously, lipid traits were modeled as a function of variables belonging to six comprehensive classes of variables: inflammation, which comprises the inflammatory biomarkers interleukin (IL)-1β, IL- 6, IL-10, C-reactive protein (CRP), monocyte chemoattractant protein 1 (MCP-1) and tumor necrosis factor-alpha (TNF-α); insulin, fasting blood glucose levels, and absence or presence of insulin resistance according to the homeostasis model assessment of insulin resistance (HOMA-IR); anthropometric characteristics (body mass index, BMI; waist circumference, and waist circumference to height ratio); socioeconomic and demographic variables (sex, age, educational attainment); systolic and diastolic blood pressure; and lifestyle characteristics (alcohol and tobacco use, diet quality and physical activity) [7]. Lipid traits were converted through rank-based normal inverse transformation to meet statistical modeling assumptions.

BMI was estimated using information of height and weight of participants, and categorized into presence or absence of overweight (including overweight and obesity), according to age group. Twelve dietary components were evaluated and combined into the Healthy Eating Index Revised and adapted for the Brazilian population (BHEI-R) to assess diet quality: dark green and orange vegetables, total vegetables, whole fruits, total fruits, legumes, whole grains, total grains, meats, eggs and legumes, milk and dairy products, saturated fat, oils, sodium, and the component corresponding to calories from solid fat, alcohol and added sugar (SoFAAS). Dietary data were obtained from two 24-hour dietary recalls, adjusted for usual intake distributions using the Multiple Source Method. The International Physical Activity Questionnaire (IPAQ)-Long Form, adapted to Portuguese and validated for the Brazilian population, was adopted for assessment of the physical activity level. Details on the phenotypic data collection and calculation of indicators are described elsewhere [17, 18].

### Genetic markers and quality control

DNA was quantified using the Qubit™ dsDNA BR DNA Quantification Kit in Qubit® 2.0 fluorometer (Thermo Fisher Scientific, Waltham, USA) from blood samples. Information from 864 free-living healthy individuals was genotyped with the Axiom™ 2.0 Precision Medicine Research Array (Affymetrix Inc, Santa Clara, CA), and 681 individuals were considered unrelated (genomic relatedness matrix, GRM, estimations > 0.125) [19]. Global ancestry was assessed with the SNPRelate package in R software v4.1.0 and PLINK 2.0 using 393,284 markers from the array that were also present in common with the 1000 Genomes Project phase 3 (1 KGP) after quality control pruning [20] (Table S1).

#### GWAS

After exclusion of individuals with missing phenotype data, a GWAS was performed for 667 unrelated individuals, with SNPs filtered based on the criteria of Hardy-Weinberg Equilibrium (P) ≥ 10^− 5^ and MAF > 0.05, using the genetic information of 330,656 SNPs. The GWAS approach used the traditional polygenic model of additive effects:

1 $${y}_{i}=\mu +{\beta }^{{\prime }}\times {X}_{i}+{{\beta }^{{\prime }}}_{{SNP}_{i}}\times {X}_{{SNP}_{i}}+{\epsilon }_{i}$$

Where *y*_*i*_ = response variable of the *i*^th^ individual; *µ* = trait mean; *β’* = transposed vector of covariate effects; *X*_*i*_ = vector of covariates; *X*_*SNPi*_ = vector with genotype information for the *i*^th^ individual; *β’*_*SNPi*_ = transposed vector of SNP effects; and *ε*_*i*_ = residual term associated with the *i*^th^ individual.

The GWASs under the linear model approach were performed using the 10^− 5^ significance level for the HDL-c, LDL-c, TGL, HDL-c/LDL-c, total cholesterol, VLDL-c, and non-HDL-c phenotypes, according to their respective selected models.

The adjustment baseline covariates age, sex, age-sex interaction, age^2^, and presence of overweight were commonly used across phenotypes in previous association analyses to avoid confounding, as in other studies [8]. The first two principal components of global ancestry (PC1 and PC2) were included to account for the highly admixed population characteristics [21–23]. The selected models with PC1, PC2 and significant covariates with association to each of the lipid traits are shown in Table 1. The synthesis of variables in the dataset are presented in Table 2.

Additionally, linkage disequilibrium analysis for the significant SNPs that were associated with more than two lipid traits and three common *FTO* SNPs (rs1421085, rs17817449 and rs9939609) was performed. Both GWAS and linkage disequilibrium analysis were performed using R 4.3.0.

**Table 1:** Selected models of serum lipid traits used for GWAS.

Lipid Trait	Covariates included for GWAS
TGL	Age, Age^2^, Sex, BMI, Insulin resistance, MCP1, SBP, PA leisure, PC1 and PC2
VLDL-c	Age, Age^2^, Sex, BMI, Insulin resistance, Smoking (current), MCP1, SBP, PA leisure, PC1 and PC2
LDL-c	Age, Age^2^, Hypolipidemic use, DBP, PC1 and PC2
HDL-c	Age^2^, BMI, TNF-α, Insulin, Smoking(current), SBP, SoFAAS, Sodium, PC1 and PC2
non-HDL-c	Age, Age^2^, BMI, Hypolipidemic drug use, Glucose, PC1 and PC2
LDL-c/HDL-c	Age, Age^2^, BMI, Hypolipidemic drug use, Glucose, CRP, SBP, PC1 and PC2
Total Chol	Age, Age^2^, Hypolipidemic drug use, Glucose, DBP, PC1 and PC2

BMI = Body Mass Index; CRP = C-reactive protein; DBP = Diastolic blood pressure; DLP adj. = Any dyslipidemia adjusted by hypolipidemic drug; MCP1 = Monocyte chemoattractant protein; PA global = Global physical activity; PA leisure = Physical activity during leisure; PC = Principal component of ancestry; SBP = Systolic blood pressure; SoFAAS = Calories obtained from added sugar, solid fat, and alcohol; TGL = triglycerides; TNF-α = Tumor necrosis factor α

**Table 2:** Descriptive statistics of the ISA-Nutrition dataset

Characteristic	Total *N* = 667*	Missing cases
Age (years)	49 (18, 64)	
Age group		
Adolescent	199 (30%)	
Adult	219 (33%)	
Older Adult	249 (37%)	
Sex		
Female	309 (46%)	
Male	358 (54%)	
Alcohol use		6
No	505 (76%)	
Yes	156 (24%)	
Smoking		4
Never	467 (70%)	
Former smoker	110 (17%)	
Smoker	86 (13%)	
Ethnicity		8
Yellow	1 (0.2%)	
White	346 (53%)	
Indigenous	2 (0.3%)	
Other	28 (4.2%)	
Brown	221 (34%)	
Black	61 (9.3%)	
Overweight		3
No	367 (55%)	
Yes	297 (45%)	
DLP adj.		
No	224 (34%)	
Yes	443 (66%)	
Insulin resistance		5
No	352 (53%)	
Yes	310 (47%)	
Glucose (mg/dL)	94 (88, 104)	1
Insulin (uui/mL)	11 (8, 16)	4
DBP (mmHg)	76 (68, 83)	4
SBP (mmHg)	125 (115, 141)	4
TNF-α (pg/mL)	11.3 (8.4, 14.3)	17
MCP1 (pg/mL)	281 (217, 349)	17
CRP (mg/L)	0.30 (0.10, 0.76)	17
PA leisure (min/week)	0 (0, 135)	11
PA global (min/week)	420 (160, 1,108)	17
Sodium	2.13 (0.82, 3.59)	6
SoFAAS	9.5 (6.3, 12.4)	6
PC1	-0.003 (-0.009, 0.003)	
PC2	-0.013 (-0.018, -0.009)	
AFR Global Ancestry	0.167 (0.035, 0.299)	
EUR Global Ancestry	0.758 (0.603, 0.929)	
AMR Global Ancestry	0.042 (0.000, 0.090)	
Total cholesterol (mg/dL)	168 (140, 199)	
TGL (mg/dL)	100 (73, 139)	
HDL-c (mg/dL)	43 (35, 52)	
LDLc (mg/dL)	101 (78, 126)	
LDL-c/HDL-c	2.38 (1.65, 3.21)	
VLDLc (mg/dL)	20 (15, 28)	
Non-HDL-c (mg/dL)	123 (96, 154)	

*Median (IQR); n (%); AFR = African; EUR = European; AMR = Native American; BMI = Body Mass Index; CRP = C-reactive protein; DBP = Diastolic blood pressure; DLP adj. = Any dyslipidemia adjusted by hypolipidemic drug; MCP1 = Monocyte chemoattractant protein; PA global = Global physical activity; PA leisure = Physical activity during leisure; PC = Principal component of ancestry; SBP = Systolic blood pressure; SoFAAS = Calories obtained from added sugar, solid fat, and alcohol; TGL = triglycerides; TNF-α = Tumor necrosis factor α

## Results

### GWAS - linear regression model

There were 19 significantly different SNPs associated with lipid traits, most of which corresponded to intron variants. Three variants (rs1562012, rs16972039, and rs73401081) and two variants (rs8025871 and rs2161683) were associated with two and three phenotypes, respectively. Non-HDL-c had the highest number of associations, as opposed to VLDL-c and LDL-c/HDL-c ratio. Among the associations, 14 and 12 had positive and negative coefficients, respectively (Table 3).

**Table 3:** SNPs significantly associated with lipid traits according to the polygenic additive model

SNP	CHR	β	*p* value	Phenotype	Gene Consequence	MAF
rs9322929	14	-0.249	8.68E-06	Total Chol	-	0.25
rs4775168	15	0.253	7.30E-06	Total Chol	*FAM81A* : Intron Variant	0.24
rs8025871	15	0.290	5.49E-07	Total Chol	*FAM81A* : Intron Variant	0.22
rs2161683	16	-0.384	3.47E-06	Total Chol	*ZFHX3* : Intron Variant	0.10
rs269029	5	-0.277	3.41E-06	HDL	*CDH12* : Intron Variant	0.29
rs4889986	17	0.559	7.53E-06	HDL	-	0.05
rs4727494	7	-0.223	9.22E-06	LDL	*COL26A1* : Intron Variant	0.36
rs2553251	8	0.222	7.35E-06	LDL	*WRN* : 500B Downstream Variant; *LOC105379358*: 2KB Upstream Variant	0.42
rs73401081	9	0.245	5.01E-07	LDL	*PTPRD* : Intron Variant	0.38
rs2890868	9	-0.222	8.19E-06	LDL	*PTPRD* : Intron Variant	0.39
rs8025871	15	0.271	4.61E-06	LDL	*FAM81A* : Intron Variant	0.22
rs2161683	16	-0.389	3.86E-06	LDL	*ZFHX3* : Intron Variant	0.10
rs16972039	16	-0.360	2.97E-06	LDL	*ZFHX3* : Intron Variant	0.12
rs6716254	2	0.251	9.97E-07	LDL/HDL	*WIPF1* : Intron Variant	0.33
rs597742	1	0.215	8.73E-06	non-HDL	*LINC02778* : Intron Variant	0.35
rs7591899	2	-0.383	8.72E-06	non-HDL	*POMC* : Intron Variant	0.09
rs1158866	4	0.226	2.25E-06	non-HDL	*LOC105374505* : Intron Variant	0.46
rs73401081	9	0.213	5.25E-06	non-HDL	*PTPRD* : Intron Variant	0.38
rs2224969	13	-0.221	7.16E-06	non-HDL	*DACH1* : Intron Variant	0.36
rs8025871	15	0.273	1.56E-06	non-HDL	*FAM81A* : Intron Variant	0.22
rs2161683	16	-0.409	4.25E-07	non-HDL	*ZFHX3* : Intron Variant	0.10
rs16972039	16	-0.368	7.06E-07	non-HDL	*ZFHX3* : Intron Variant	0.12
rs3737369	18	-0.333	5.44E-06	non-HDL	*ENOSF1* : Intron Variant	0.13
rs1562012	2	0.405	3.39E-06	VLDL	-	0.07
rs1562012	2	0.392	6.43E-06	TGL	-	0.07
rs76918426	11	0.441	5.05E-06	TGL	-	0.06

CHR = Chromosome; MAF = Minor allele frequency; SNP = Single nucleotide polymorphism

**Fig. 1:**
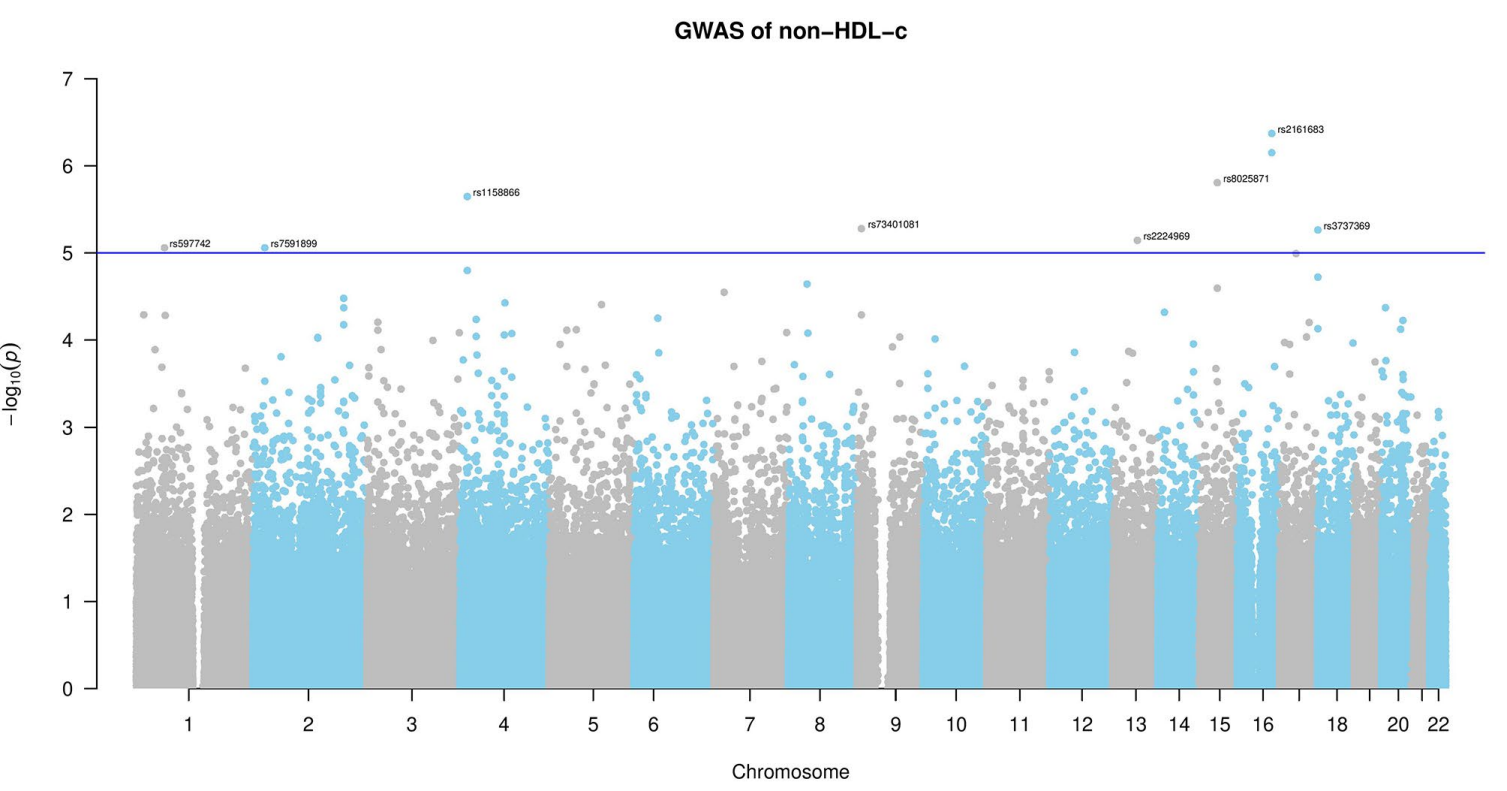
Manhattan plot of the significant SNPs associated with non-HDL-c

Manhattan plots with SNPs above the significance threshold are shown in Fig. 1 and Figures S1-S6.

## Discussion

The GWAS under the polygenic additive model revealed 19 novel significant associations between SNPs and lipid traits in the present study. Some of the associations were consistently found across two to three lipid traits, which is in line with the well-established understanding of the metabolism and physiology of lipoproteins. The literature on specific associations of phenotypes with SNPs identified in the present study showed that only rs7591899 was previously investigated in relation to glucometabolic traits, which presented conflicting evidence [24, 25].

A recent GWAS performed through Mendelian randomization to evaluate circulating lipoproteins, including HDL, LDL, and triglycerides levels, using data from the UKBiobank, identified more than one thousand associated SNPs. However, none of their results were replicated in the present investigation [26]. Similarly, findings from other GWASs that included data from underrepresented populations also lacked correspondence with the present results [27–30]. While a recent multi-ancestry meta-analysis incorporated a sample from the Brazilian population, its focus was on exploring associations between the interaction effects of LDL-c, HDL-c, and triglycerides with physical activity, rather than solely assessing the lipid profiles independently [31]. Also, there was no correspondence between the results of that study (significant SNPs on *CLASP1*, *LHX1*, *SNTA1*, and *CNTNAP2* genes) and the ones of the present investigation. Hence, it should be noted that findings from these studies should not be directly compared due to several methodological differences, including sample size, evaluated trait, genetic ancestry, genotyping platform, and significance level, among others.

The results of the present study showed that phenotypic lipid traits were significantly associated with SNPs linked to the genes *CDH12*, *COL26A1*, *DACH1*, *ENOSF1*, *FAM81A*, *LINC02778*, *LOC105374505*, *LOC105379358*, *POMC*, *PTPRD*, *WIPF1*, *WRN*, and *ZFHX3*. Some genes have been previously investigated due to links with lipid metabolism (*FAM81A*) [32], low-density lipoprotein cholesterol and obesity (*ZFHX3*) [33, 34], myocardial infarction (*CDH12*) [35], nonalcoholic fatty liver disease (*PTPRD*) [36], cardioembolic stroke risk (*WIPF1*) [37], satiety and obesity (*POMC*), fasting lipids and insulin in children (*POMC*) [38], and atherosclerosis (DACH1) [39].

In the present study, the majority of the variants linked to two or more phenotypes were present in intronic regions, particularly within the genes *FAM81A, ZFHX3, PTPRD*, and *POMC*. Except for *POMC* (proopiomelanocortin), there were two variants found for each of the genes, which suggested that the significant variants within a given gene might be in linkage disequilibrium (LD) with each other. This was confirmed by additional LD analysis, which showed that *ZFHX3* SNPs and *FAM81A* were in strong and weak LD, respectively, while *PTPRD* SNPs were in linkage equilibrium (Table S2 and S3).

*POMC* is responsible for encoding a preproprotein subjected to extensive, tissue-specific, post translational processing, resulting in up to ten possible different active peptides involved in several cellular processes. One of the main peptides is lipotropin beta, which is responsible for the mobilization of fat from adipose tissue [40]. Variants in *POMC* have been linked to obesity and hyperphagia, likely through (a) leptin-dependent sympathetic innervation of adipose tissue, which then decreases the mobilization of lipids within the white adipose tissue (WAT), and (b) impaired MC4R signaling in the hypothalamus because of the lack of α-MSH and diacetyl-α-MSH, which leads to increased appetite [41–43].

Regarding *FAM81A*, there was no function assigned for either rs4775168 or rs8025871, being the latter linked to both LDL-c and non-HDL-c. However, it should be noted that rs8025871 is near the rs17302400 variant within the same gene, which has been previously associated with visceral adipose tissue [44]. In a previous GWAS performed on multiple ancestry participants from the Million Veteran Program, variants in other FAM genes were shown to be associated with several lipid traits, e.g., *FAM13A* with HDL-c; *FAM136A* with both LDL-c and total cholesterol; and *FAM117B* with both LDL-c and total cholesterol [28]. In addition, an association with *FAM241B* was detected in a study with a smaller sample of the underrepresented Indian population [29].

Furthermore, the two variants in *ZFHX3*, which encodes the zinc-finger homeobox 3 protein are present in intronic regions and have not been described in other studies. Nonetheless, the ZFHX3 gene acts as a transcription regulator and some of its polymorphisms were associated with risk of atrial fibrillation [45, 46]. Considering that *ZFHX3* is located on chromosome 16, the same chromosome in which several SNPs in the *FTO* obesity-related gene are found, a possible hypothesis for the significant associations identified is that they might be in linkage disequilibrium with *FTO* and *FTO-*related genes [47].

For instance, in comparison to *FTO*, *ZFHX3* has approximately 1 million base-pairs closer to Iroquois homeobox protein 3 (*IRX3*), which is known to mechanistically interact with the genetic variation of *FTO* to influence obesity and related metabolic disorders [48]. Importantly, the effects have also been observed in admixed Latin populations and might be connected with hepatic lipid metabolism, as shown by negative correlations of the transcription factor with serum triglycerides, LDL-c, uric acid, and total cholesterol levels [49, 50]. This hypothesis was confirmed in this study, as shown by low, albeit significant LD values between the *ZFHX3* SNPs and three main SNPs in the *FTO* gene (rs1421085, rs17817449 and rs9939609) showing very low LD values (Table S2 and S3).

Concerning *PTPRD* (protein tyrosine phosphatase receptor type D), neither of the two variants had been associated with lipid traits in previous studies, and, accordingly, its gene product, which is a signaling peptide involved in several cellular processes, has no reported involvement in lipid metabolism.

Major part of the significant associations with single phenotypes were in genes that have broader ranges of cellular functions (e.g., cell adhesion, cell growth, differentiation, organization of cytoskeleton), with no sound implication for lipid metabolism or any cardiometabolic-related outcome. Notably, there were pinpointed variants in two noncoding RNA (ncRNA) genes (LOC105374505 and LINC02778), that have not been characterized thus far. It is widely recognized that ncRNAs have important regulatory functions in several diseases and health conditions, including cancer, metabolic disorders, diabetes, and inflammation [51, 52].

Interestingly, a novel ncRNA has been reported to reprogram lipid metabolism, leading to the accumulation of lipids inside the cell and promoting hepatocellular carcinoma progression [53]. However, the roles of the ncRNAs in the onset of dyslipidemia or other phenotypes in the Brazilian population has yet to be determined by further investigation.

Furthermore, the novel evidence identified in the present study may contribute to advances in precision medicine applied for treatment of cardiometabolic diseases, including dyslipidemia, and metabolic syndrome. The identification of genetic features linked to lipid traits may support pharmacogenomic investigations for the prediction of treatment responses, allowing to avoid adverse effects and improve therapies through integrated approaches for dyslipidemia at the individual level, in addition to supporting disease prevention strategies that may reduce treatment costs in national health systems [54–56].

### Study strengths and limitations

The study presents numerous strengths. The GWAS was performed in a Brazilian cohort of free-living individuals from a study with a sample that is representative at population level in the largest city of the country, adopting strict methodological rigor regarding data collection and analysis. In addition, the population evaluated has admixed ancestries and is underrepresented in genetic research, which may contribute to the understanding of genes and lipid related outcomes, considering that the availability of numerous GWASs in multi ancestries populations may contribute to research progress in this field with the ultimate goal of improving lipid profiles and reducing CVD risk [57].

Importantly, certain limitations should be considered in the interpretation of the aforementioned results. First, the dataset had a small sample size, which decreases the study power for detection of significant associations. Second, the genetic data included a limited set of SNP genotype data, which might lack information on other important markers with possible clinical relevance. Third, there was lack of specific information on other lipids, like LDL-c fractions and apolipoproteins usually associated with risk for CVD (e.g., apoB48, apoB100, apoC-III). Finally, the use of cross-sectional data imposes challenges for interpretation of the clinical significance of SNPs using data from a single population due to limitations in the establishment of causality; thus, additional research is required on the associations between SNPs and lipid profiles identified in the present study.

## Conclusions

The GWAS results offer insights regarding the genetic structure underlying lipid traits in an underrepresented population with high ancestry admixture. The associations identified in the study were robust across multiple lipid phenotypes, and some of the associations were significant for two or more variants. Furthermore, the findings raise important questions about the role of ncRNAs in lipid metabolism, which remains a relatively unexplored subject.

Nevertheless, comparisons with other populations should be approached with caution, and further replication on larger datasets and in other populations with admixed backgrounds should be rendered. Thus, the present findings may guide follow-up investigations aiming at replicating the results, and to enhance interpretability by identifying credible or causal variants involved in the metabolism of lipoproteins, which may facilitate the identification of novel targets for therapies that improve lipid profile. Further evidence may be achieved using fine-mapping, functional annotation, and causal inference approaches, as well as candidate-gene experiments focused on the genes *FAM81A, ZFHX3*, *PTPRD*, and *POMC*.

### Electronic supplementary material

Below is the link to the electronic supplementary material.

Table S1. SNPs pruning for quality control of ancestry analysis of 681 uncorrelated individuals, 2015 ISA-Nutrition

Figure S1: Manhattan plot of the significant SNPs associated with LDL-c

Figure S2: Manhattan plot of the significant SNPs associated with HDL-c

Figure S3: Manhattan plot of the significant SNPs associated with VLDL-c

Figure S4: Manhattan plot of the significant SNPs associated with LDL-c/HDL-c

Figure S5: Manhattan plot of the significant SNPs associated with total cholesterol

Figure S6: Manhattan plot of the significant SNPs associated with triglycerides

Supplementary Material 8

Table S2: Correlation coefficients of linkage disequilibrium analysis between SNPs significantly associated with two or more lipid traits and SNPs in the FTO gene

Table S3: *P*-values of linkage disequilibrium analysis between SNPs significantly associated with two or more lipid traits and SNPs in the FTO gene

## Data Availability

The datasets analyzed in the current study are available upon reasonable request from the corresponding author.

## Abbreviations

CRP: C-reactive protein
CVD: Cardiovascular diseases
DLP: Dyslipidemia
GRM: Genomic relatedness matrix
GWAS: Genome-Wide Association Study
HDL-c: High-density lipoprotein cholesterol
HOMA-IR: Homeostatic Model Assessment for Insulin Resistance
IL: Interleukin
IPAQ: International Physical Activity Questionnaire
ISA Nutrition: Health Survey of São Paulo with Focus on Nutrition
LDL-c: Low-density lipoprotein cholesterol
MCP-1: Monocyte chemoattractant protein 1
ncRNA: Noncoding ribonucleic acid
PA: Physical activity
PC: Principal component
SNP: Single nucleotide polymorphism
SoFAAS: Consumption of calories from solid fat, alcohol and added sugar
TGL: Triglycerides
TNF-α: Tumor necrosis factor alpha
VLDL-c: Very low-density lipoprotein cholesterol
WAT: White adipose tissue

## References

[1] World Health Organization (WHO). Global status report on noncommunicable diseases 2014. Geneva, World Health Organization;; 2014. https://www.who.int/publications/i/item/9789241564854. Accessed on 15 May 2023.

[2] Pogosova N. Costs associated with cardiovascular disease create a significant burden for society and they seem to be globally underestimated. Eur J Prev Cardiol. 2019;26(11):1147–9. 10.1177/2047487319842578. 30955346 10.1177/2047487319842578

[3] Lin H-Q, Wu J-Y, Chen M-L, Chen F-Q, Liao Y-J, Wu Y-T, et al. Prevalence of dyslipidemia and prediction of 10-year CVD risk among older adults living in southeast coastal regions in China: a cross-sectional study. Clin Interv Aging. 2019;14:1119–29. 10.2147/CIA.S207665. 31354254 10.2147/CIA.S207665 PMC6590841

[4] Sascău R, Clement A, Radu R, Prisacariu C, Stătescu C. Triglyceride-rich lipoproteins and their remnants as silent promoters of atherosclerotic cardiovascular disease and other metabolic disorders: a review. Nutrients. 2021;13(6):1774. 10.3390/nu13061774. 34067469 10.3390/nu13061774 PMC8224751

[5] Willer CJ. Discovery and refinement of loci associated with lipid levels. Nat Genet. 2013;45(11):1274–83. 10.1038/ng.2797. 24097068 10.1038/ng.2797 PMC3838666

[6] Cadby G, Melton PE, McCarthy NS, Giles C, Mellett NA, Huynh K, et al. Heritability of 596 lipid species and genetic correlation with cardiovascular traits in the Busselton Family Heart Study. J Lipid Res. 2020;61(4):537–45. 10.1194/jlr.RA119000594. 32060071 10.1194/jlr.RA119000594 PMC7112151

[7] Leite JMRS, Pereira JL, Damasceno NRT, Soler JMP, Fisberg RM, Rogero MM, et al. Association of dyslipidemia with single nucleotide polymorphisms of the cholesteryl ester transfer protein gene and cardiovascular disease risk factors in a highly admixed population. Clin Nutr ESPEN. 2023;58:242–52. 10.1016/j.clnesp.2023.10.002. 38057013 10.1016/j.clnesp.2023.10.002

[8] Graham SE, Clarke SL, Wu K-HH, Kanoni S, Zajac GJM, Ramdas S, et al. The power of genetic diversity in genome-wide association studies of lipids. Nature. 2021;600:675–9. 10.1038/s41586-021-04064-3. 34887591 10.1038/s41586-021-04064-3 PMC8730582

[9] Lin M, Park DS, Zaitlen NA, Henn BM, Gignoux CR. Admixed populations improve power for variant discovery and portability in genome-wide association studies. Front Genet. 2021;12. 10.3389/fgene.2021.673167. 10.3389/fgene.2021.673167 PMC8181458 34108994

[10] de Oliveira CM, Pereira AC, de Andrade M, Soler JM, Krieger JE. Heritability of cardiovascular risk factors in a Brazilian population: Baependi Heart Study. BMC Med Genet. 2008;9(1):32. 10.1186/1471-2350-9-32. 18430212 10.1186/1471-2350-9-32 PMC2386446

[11] Oki E, Norde MM, Carioca AAF, Ikeda RE, Souza JMP, Castro IA, et al. Interaction of SNP in the CRP gene and plasma fatty acid profile in inflammatory pattern: a cross-sectional population-based study. Nutrition. 2016;32(1):88–94. 10.1016/j.nut.2015.07.015. 26456189 10.1016/j.nut.2015.07.015

[12] Crews DE, Kamboh MI, Mancilha-Carvalho JJ, Kottke B. Population genetics of apolipoprotein A-4, E, and H polymorphisms in Yanomami indians of northwestern Brazil: associations with lipids, lipoproteins, and carbohydrate metabolism. Hum Biol. 1993;65(2):211–24. 8449482

[13] Moriguchi Watanabe L, Bueno AC, de Lima LF, Ferraz-Bannitz R, Dessordi R, Guimarães MP, et al. Genetically determined variations of selenoprotein P are associated with antioxidant, muscular, and lipid biomarkers in response to Brazil nut consumption by patients using statins. Br J Nutr. 2022;127(5):679–86. 10.1017/s000711452100146x. 33947487 10.1017/s000711452100146x

[14] Fisberg R, Sales C, Fontanelli M, Pereira J, Alves M, Escuder M, et al. 2015 Health Survey of São Paulo with Focus in Nutrition: rationale, design, and procedures. Nutrients. 2018;10(2):169. 10.3390/nu10020169. 29389885 10.3390/nu10020169 PMC5852745

[15] David S. A current guide to candidate gene association studies. Trends Genet. 2021;37(12):1056–9. 10.1016/j.tig.2021.07.009. 34400010 10.1016/j.tig.2021.07.009

[16] Pereira JL, Vieira DA, dos S, Alves MCGP, César CLG, Goldbaum M, Fisberg RM. Excess body weight in the city of São Paulo: panorama from 2003 to 2015, associated factors and projection for the next years. BMC Public Health. 2018;18(1):1332. 10.1186/s12889-018-6225-8. 30509223 10.1186/s12889-018-6225-8 PMC6276135

[17] IPAQ Research Committee. Scoring protocol for the International Physical Activity Questionnaire (IPAQ). 2005. Available from: https://sites.google.com/view/ipaq/score. Accessed on 15 May 2023.

[18] Previdelli ÁN, de Andrade SC, Pires MM, Ferreira SRG, Fisberg RM, Marchioni DM. A revised version of the healthy eating index for the Brazilian population. Rev Saude Publica. 2011;45(4):794–8. 10.1590/S0034-89102011005000035. 21655703 10.1590/S0034-89102011005000035

[19] Thermo Fisher Scientific. Axiom Genotyping Solution data analysis user guide. 2020. Available from: https://assets.thermofisher.com/TFS-Assets/LSG/manuals/axiom_genotyping_solution_analysis_guide.pdf. Accessed on 28 December 2023.

[20] Zheng X, Levine D, Shen J, Gogarten SM, Laurie C, Weir BS. A high-performance computing toolset for relatedness and principal component analysis of SNP data. Bioinformatics. 2012;28(24):3326–8. 10.1093/bioinformatics/bts606. 23060615 10.1093/bioinformatics/bts606 PMC3519454

[21] Coelho AVC, Moura RR, Cavalcanti CAJ, Guimarães RL, Sandrin-Garcia P, Crovella S, et al. A rapid screening of ancestry for genetic association studies in an admixed population from Pernambuco, Brazil. Genet Mol Res. 2015;14(1):2876–84. 10.4238/2015.March.31.18. 25867437 10.4238/2015.March.31.18

[22] Pena SDJ, Santos FR, Tarazona-Santos E. Genetic admixture in Brazil. Am J Med Genet Part C Semin Med Genet. 2020;184(4):928–38. 10.1002/ajmg.c.31853. 33205899 10.1002/ajmg.c.31853

[23] de Andrade M, Ray D, Pereira AC, Soler JP. Global individual ancestry using principal components for family data. Hum Hered. 2015;80(1):1–11. 10.1159/000381908. 26159893 10.1159/000381908 PMC4583840

[24] Sharma NK, Comeau ME, Montoya D, Pellegrini M, Howard T, Langefeld CD, et al. Integrative analysis of glucometabolic traits, adipose tissue DNA methylation, and gene expression identifies epigenetic regulatory mechanisms of insulin resistance and obesity in African americans. Diabetes. 2020;69(12):2779–93. 10.2337/db20-0117. 32928872 10.2337/db20-0117 PMC7679782

[25] Cadena López RO, Soto Ontiveros VJ, Aguilar Galarza BA, Anaya Loyola MA, García Gasca T, García Muñoz W et al. Asociación de variantes genéticas de MC4R, PCSK1 y POMC a obesidad. Revista Nthe. 2022; Edición especial:28–35.

[26] Richardson TG, Sanderson E, Palmer TM, Ala-Korpela M, Ference BA, Davey Smith G, et al. Evaluating the relationship between circulating lipoprotein lipids and apolipoproteins with risk of coronary heart disease: a multivariable mendelian randomisation analysis. PLOS Med. 2020;17(3):e1003062. 10.1371/journal.pmed.1003062. 32203549 10.1371/journal.pmed.1003062 PMC7089422

[27] Bentley AR, Sung YJ, Brown MR, Winkler TW, Kraja AT, Ntalla I, et al. Multi-ancestry genome-wide gene-smoking interaction study of 387,272 individuals identifies new loci associated with serum lipids. Nat Genet. 2019;51(4):636–48. 10.1038/s41588-019-0378-y. 30926973 10.1038/s41588-019-0378-y PMC6467258

[28] Klarin D, Damrauer SM, Cho K, Sun YV, Teslovich TM, Honerlaw J, et al. Genetics of blood lipids among ~ 300,000 multi-ethnic participants of the million veteran program. Nat Genet. 2018;50(11):1514–23. 10.1038/s41588-018-0222-9. 30275531 10.1038/s41588-018-0222-9 PMC6521726

[29] Bandesh K, Prasad G, Giri AK, Kauser Y, Upadhyay M, Basu A, et al. Genome-wide association study of blood lipids in indians confirms universality of established variants. J Hum Genet. 2019;64(6):573–87. 10.1038/s10038-019-0591-7. 30911093 10.1038/s10038-019-0591-7

[30] Wu Y, Marvelle AF, Li J, Croteau-Chonka DC, Feranil AB, Kuzawa CW, et al. Genetic association with lipids in filipinos: waist circumference modifies an APOA5 effect on triglyceride levels. J Lipid Res. 2013;54(11):3198–205. 10.1194/jlr.P042077. 24023260 10.1194/jlr.P042077 PMC3793624

[31] Kilpeläinen TO, Bentley AR, Noordam R, Sung YJ, Schwander K, Winkler TW, et al. Multi-ancestry study of blood lipid levels identifies four loci interacting with physical activity. Nat Commun. 2019;10:376. 10.1038/s41467-018-08008-w. 30670697 10.1038/s41467-018-08008-w PMC6342931

[32] Ke J, Gao W, Wang B, Cao W, Lv J, Yu C, et al. Exploring the genetic association between obesity and serum lipid levels using bivariate methods. Twin Res Hum Genet. 2022;25(6):234–44. 10.1017/thg.2022.39. 36606461 10.1017/thg.2022.39

[33] Martin R, Koref MS, Owens A, Keavney B. 180 genetic variation associated with low-density lipoprotein cholesterol levels influences ZFHX3 expression. Heart. 2013;99:A102–3. 10.1136/heartjnl-2013-304019.180. 10.1136/heartjnl-2013-304019.180

[34] Yang S-A. Association study between ZFHX3 gene polymorphisms and obesity in Korean population. J Exerc Rehabil. 2017;13(4):491–4. 10.12965/jer.1735080.540. 29114518 10.12965/jer.1735080.540 PMC5667630

[35] Derda AA, Woo CC, Wongsurawat T, Richards M, Lee CN, Kofidis T, et al. Gene expression profile analysis of aortic vascular smooth muscle cells reveals upregulation of cadherin genes in myocardial infarction patients. Physiol Genomics. 2018;50(8):648–57. 10.1152/physiolgenomics.00042.2017. 29775430 10.1152/physiolgenomics.00042.2017

[36] Chen Y, Du X, Kuppa A, Feitosa MF, Bielak LF, O’Connell JR, et al. Genome-wide association meta-analysis identifies 17 loci associated with nonalcoholic fatty liver disease. Nat Genet. 2023;55:1640–50. 10.1038/s41588-023-01497-6. 37709864 10.1038/s41588-023-01497-6 PMC10918428

[37] Gallego-Fabrega C, Muiño E, Cárcel-Márquez J, Llucià-Carol L, Lledós M, Martín-Campos JM, et al. Genome-wide studies in ischaemic stroke: are genetics only useful for finding genes? Int J Mol Sci. 2022;23:6840. 10.3390/ijms23126840. 35743317 10.3390/ijms23126840 PMC9224543

[38] Candler T, Kühnen P, Prentice AM, Silver M. Epigenetic regulation of POMC; implications for nutritional programming, obesity and metabolic disease. Front Neuroendocrinol. 2019;54:100773. 10.1016/j.yfrne.2019.100773. 31344387 10.1016/j.yfrne.2019.100773

[39] Wang Y, Wang T, Luo Y, Jiao L. Identification markers of carotid vulnerable plaques: an update. Biomolecules. 2022;12:1192. 10.3390/biom12091192. 36139031 10.3390/biom12091192 PMC9496377

[40] Smyth DG. 60 years of POMC: lipotropin and beta-endorphin: a perspective. J Mol Endocrinol. 2016;56(4):T13–25. 10.1530/JME-16-0033. 26903509 10.1530/JME-16-0033

[41] Zemel MB, Shi H. Pro-opiomelanocortin (POMC) deficiency and peripheral melanocortins in obesity. Nutr Rev. 2009;58(6):177–80. 10.1111/j.1753-4887.2000.tb01857.x. 10.1111/j.1753-4887.2000.tb01857.x 10885325

[42] van der Valk ES, Kleinendorst L, Delhanty PJD, van der Voorn B, Visser JA, van Haelst MM, et al. Obesity and hyperphagia with increased defective ACTH: a novel POMC variant. J Clin Endocrinol Metab. 2022;107(9):e3699–704. 10.1210/clinem/dgac342. 35737586 10.1210/clinem/dgac342 PMC9797039

[43] Wang P, Loh KH, Wu M, Morgan DA, Schneeberger M, Yu X, et al. A leptin-BDNF pathway regulating sympathetic innervation of adipose tissue. Nature. 2020;583(7818):839–44. 10.1038/s41586-020-2527-y. 32699414 10.1038/s41586-020-2527-y

[44] Fox CS, Liu Y, White CC, Feitosa M, Smith AV, Heard-Costa N et al. Genome-Wide Association for Abdominal Subcutaneous and Visceral Adipose Reveals a Novel Locus for Visceral Fat in Women. Bray M, editor. PLoS Genet. 2012; 8 (5):e1002695; 10.1371/journal.pgen.1002695. 10.1371/journal.pgen.1002695 PMC3349734 22589738

[45] Sakata N, Kaneko S, Ikeno S, Miura Y, Nakabayashi H, Dong X-Y, et al. TGF- β signaling cooperates with AT motif-binding Factor-1 for repression of the α -Fetoprotein promoter. J Signal Transduct. 2014;2014:1–11. 10.1155/2014/970346. 10.1155/2014/970346 PMC4106063 25105025

[46] Wei Y, Wang L, Lin C, Xie Y, Bao Y, Luo Q, et al. Association between the rs2106261 polymorphism in the zinc finger homeobox 3 gene and risk of atrial fibrillation. Medicine. 2021;100(49):e27749. 10.1097/md.0000000000027749. 34889223 10.1097/md.0000000000027749 PMC8663867

[47] Tan L-J, Zhu H, He H, Wu K-H, Li J, Chen X-D, et al. Replication of 6 obesity genes in a meta-analysis of Genome-Wide Association Studies from diverse ancestries. PLoS ONE. 2014;9(5):e96149. 10.1371/journal.pone.0096149. 24879436 10.1371/journal.pone.0096149 PMC4039436

[48] Smemo S, Tena JJ, Kim K-H, Gamazon ER, Sakabe NJ, Gómez-Marín C, et al. Obesity-associated variants within FTO form long-range functional connections with IRX3. Nature. 2014;507(7492):371–5. 10.1038/nature13138. 24646999 10.1038/nature13138 PMC4113484

[49] Ruiz Díaz MS, Mena-Yi D, Gómez- Camargo D, Mora-García GJ. Interaction analysis of FTO and IRX3 genes with obesity and related metabolic disorders in an admixed latin American population: a possible risk increases of body weight excess. Colomb Med. 2022;53(2):e2044874. 10.25100/cm.v53i2.4874. 10.25100/cm.v53i2.4874 PMC9651168 36415696

[50] Ma Y, Chen G, Yi J, Li Q, Tan Z, Fan W, et al. IRX3 plays an important role in the pathogenesis of metabolic-associated fatty liver disease by regulating hepatic lipid metabolism. Front Endocrinol. 2022;13. 10.3389/fendo.2022.895593. 10.3389/fendo.2022.895593 PMC9360787 35957832

[51] Jacovetti C, Bayazit MB, Regazzi R. Emerging classes of small non-coding RNAs with potential implications in diabetes and associated metabolic disorders. Front Endocrinol. 2021;12. 10.3389/fendo.2021.670719. 10.3389/fendo.2021.670719 PMC8142323 34040585

[52] Zhang P, Wu W, Chen Q, Chen M. Non-coding RNAs and their integrated networks. J Integr Bioinform. 2019;16(3). 10.1515/jib-2019-0027. 10.1515/jib-2019-0027 PMC6798851 31301674

[53] Xu K, Xia P, Gongye X, Zhang X, Ma S, Chen Z, et al. A novel lncRNA RP11-386G11.10 reprograms lipid metabolism to promote hepatocellular carcinoma progression. Mol Metab. 2022;63:101540. 10.1016/j.molmet.2022.101540. 35798238 10.1016/j.molmet.2022.101540 PMC9287641

[54] Hu C, Jia W. Multi-omics profiling: the way toward precision medicine in metabolic diseases. J Mol Cell Biol. 2021;13(8):576–93. 10.1093/jmcb/mjab051. 34406397 10.1093/jmcb/mjab051 PMC8697344

[55] Babu M, Snyder M. Multi-omics profiling for health. Mol Cell Proteom. 2023;22(6):100561. 10.1016/j.mcpro.2023.100561. 10.1016/j.mcpro.2023.100561 PMC10220275 37119971

[56] Alfonsi JE, Hegele RA, Gryn SE. Pharmacogenetics of lipid-lowering agents: precision or indecision medicine? Curr Atheroscler Rep. 2016;18:24. 10.1007/s11883-016-0573-6. 26993470 10.1007/s11883-016-0573-6

[57] Hou K, Bhattacharya A, Mester R, Burch KS, Pasaniuc B. On powerful GWAS in admixed populations. Nat Genet. 2021;53(12):1631–3. 10.1038/s41588-021-00953-5. 34824480 10.1038/s41588-021-00953-5 PMC8939372

