# Supplementary figures and images for: Novel loci linked to serum lipid traits are identified in a genome-wide association study of a highly admixed Brazilian population - the 2015 ISA Nutrition
Source: Lipids Health Dis. 2024 Jul 26;23:229. doi: 10.1186/s12944-024-02085-1 (PMC11282745; doi:10.1186/s12944-024-02085-1)

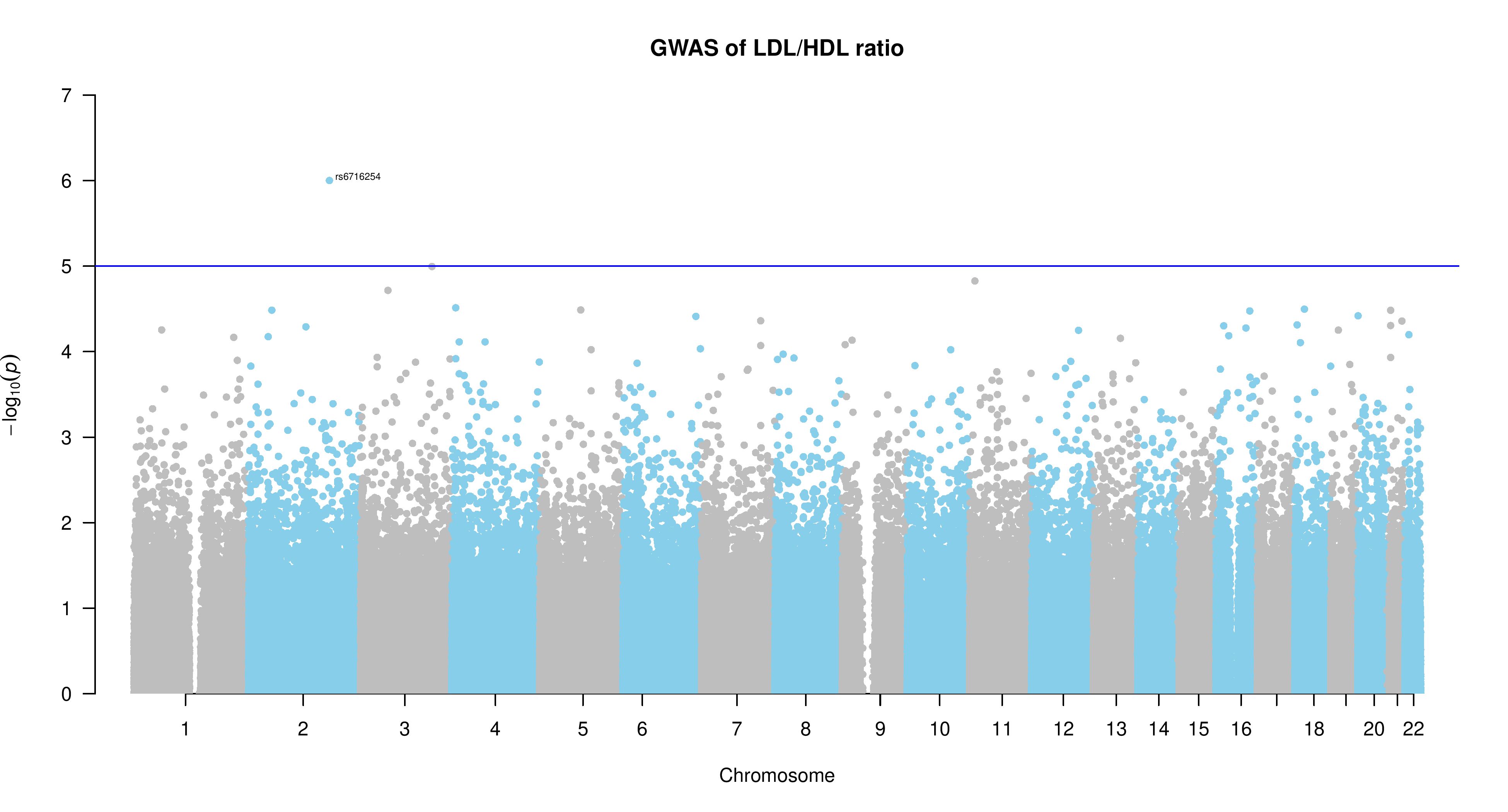

Supplement: Supplementary file 2 — Figure S1: Manhattan plot of the significant SNPs associated with LDL-c [file 12944_2024_2085_MOESM2_ESM.jpg]

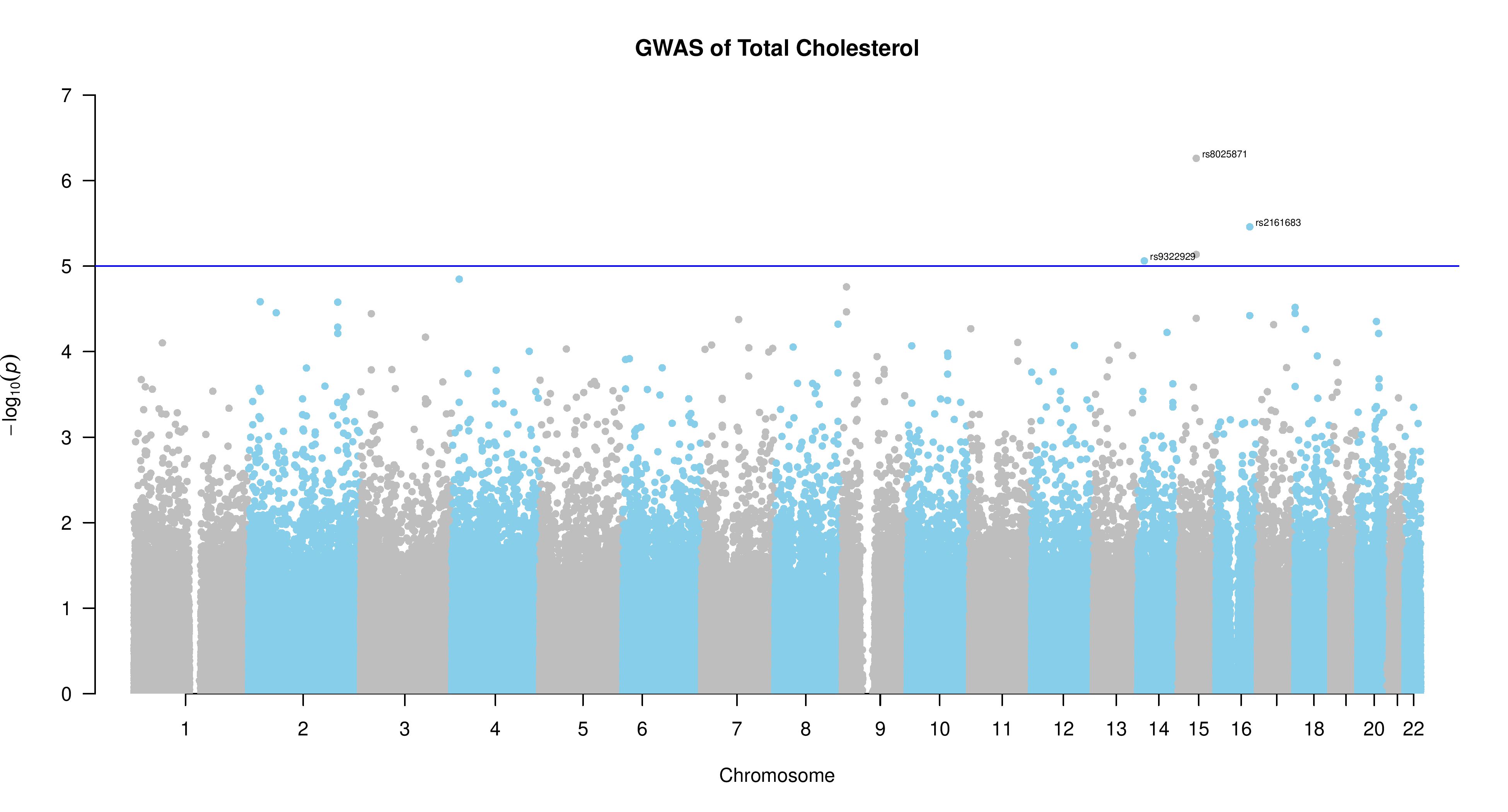

Supplement: Supplementary file 3 — Figure S2: Manhattan plot of the significant SNPs associated with HDL-c [file 12944_2024_2085_MOESM3_ESM.jpg]

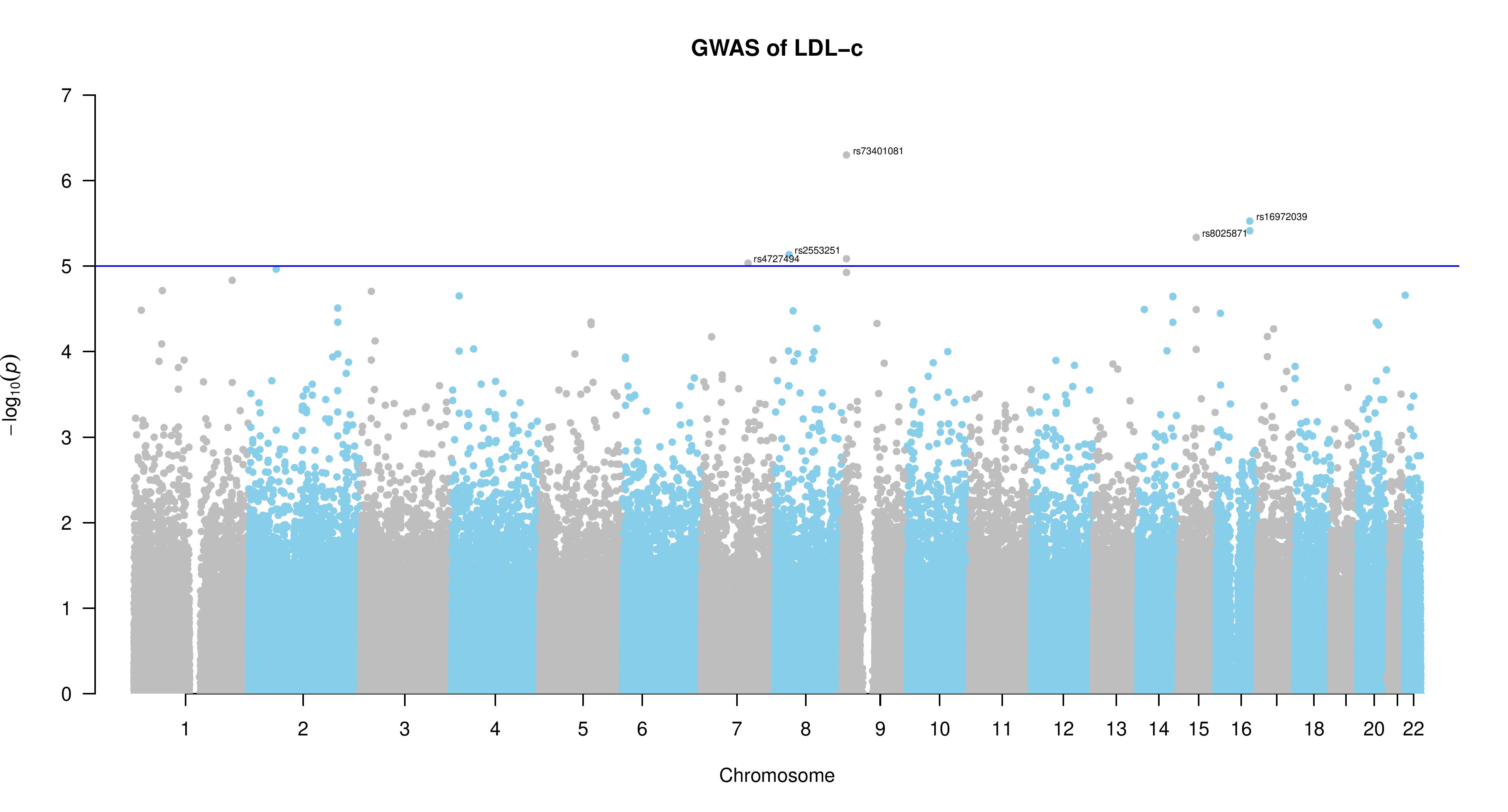

Supplement: Supplementary file 4 — Figure S3: Manhattan plot of the significant SNPs associated with VLDL-c [file 12944_2024_2085_MOESM4_ESM.jpg]

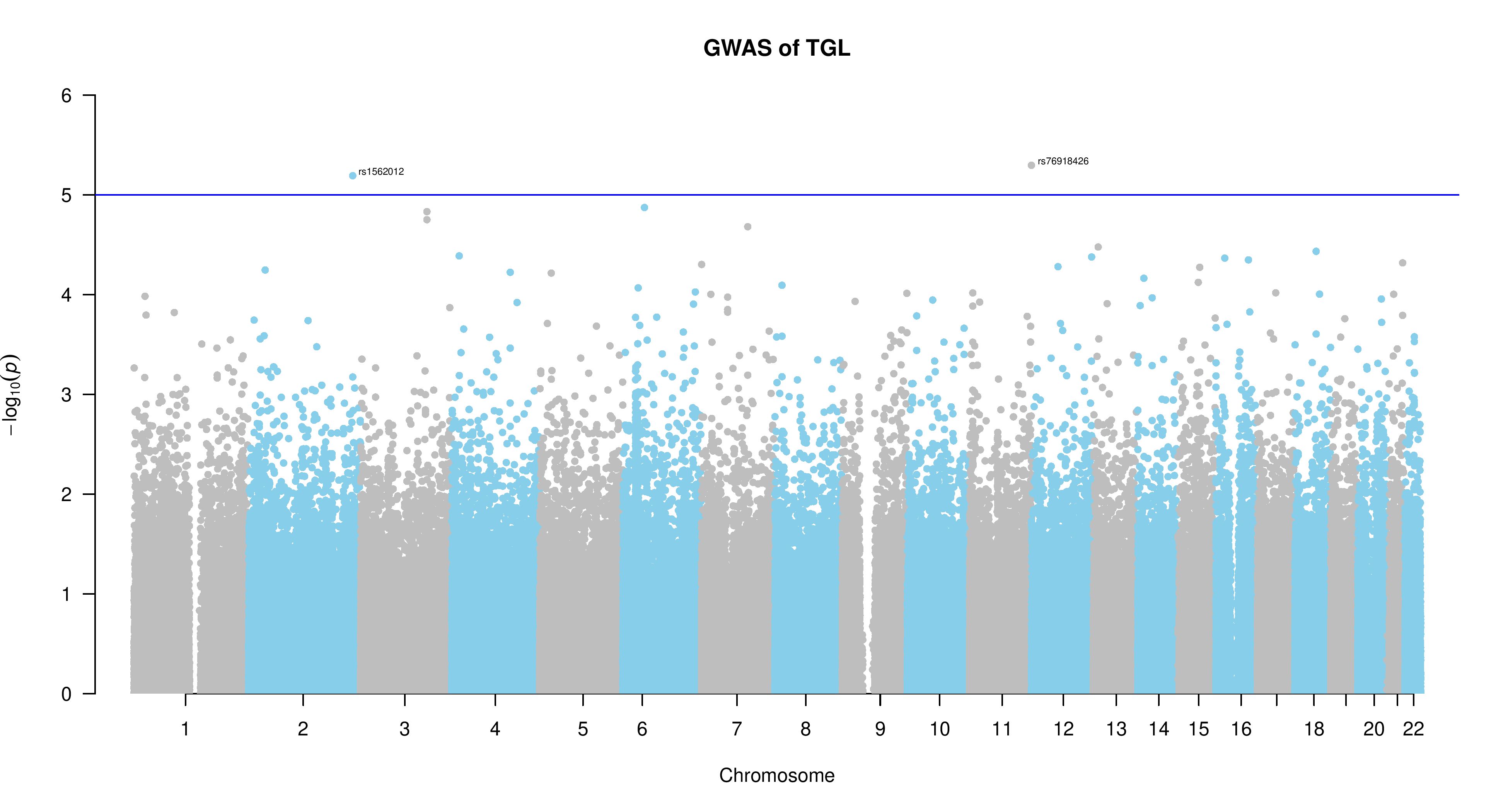

Supplement: Supplementary file 5 — Figure S4: Manhattan plot of the significant SNPs associated with LDL-c/HDL-c [file 12944_2024_2085_MOESM5_ESM.jpg]

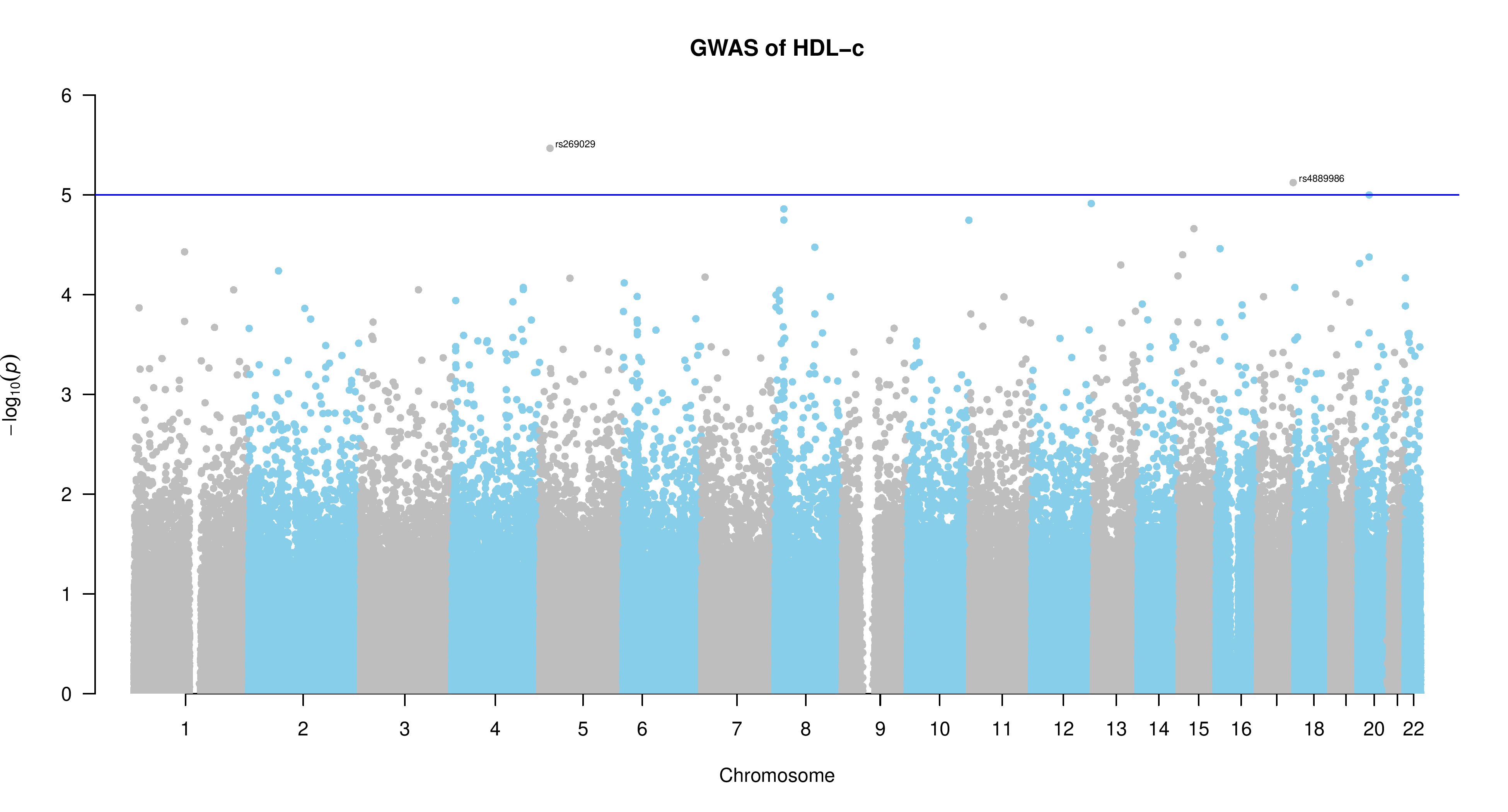

Supplement: Supplementary file 6 — Figure S5: Manhattan plot of the significant SNPs associated with total cholesterol [file 12944_2024_2085_MOESM6_ESM.jpg]

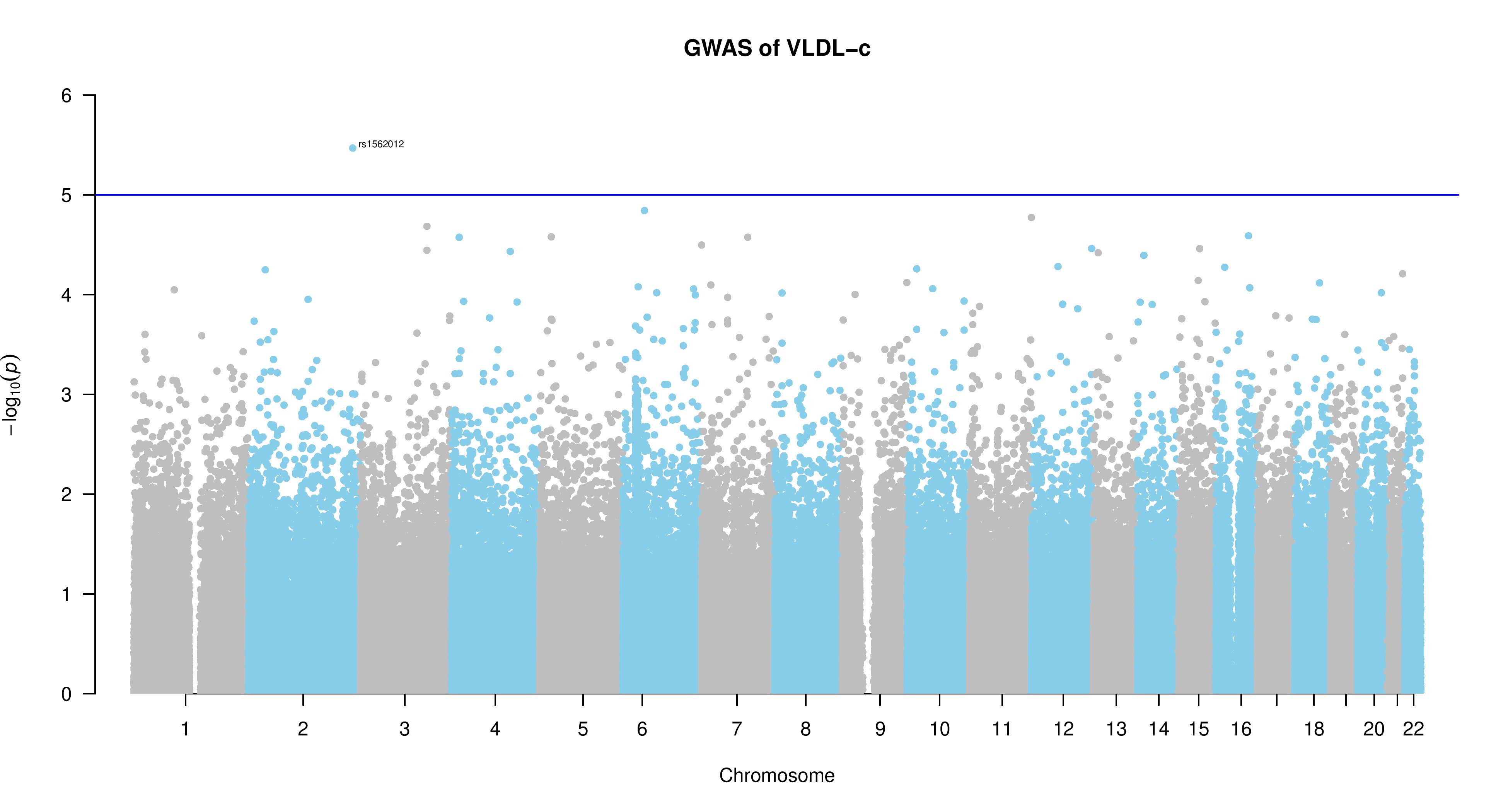

Supplement: Supplementary file 7 — Figure S6: Manhattan plot of the significant SNPs associated with triglycerides [file 12944_2024_2085_MOESM7_ESM.jpg]
